# Influence of combined physical and cognitive training on cognition: a systematic review

**DOI:** 10.1186/s12877-016-0315-1

**Published:** 2016-07-18

**Authors:** Andreas Lauenroth, Anestis E. Ioannidis, Birgit Teichmann

**Affiliations:** Network Aging Research (NAR), University of Heidelberg, Germany, Bergheimer Straße 20, 69115 Heidelberg, Germany

**Keywords:** Dual task, Combined exercise, Motor-cognitive training, Cognitive performance, Aging

## Abstract

**Background:**

Numerous daily activities require simultaneous application of motor and cognitive skills (dual-tasking). The execution of such tasks is especially difficult for the elderly and for people with (neuro-) degenerative disorders. Training of physical and cognitive abilities helps prevent or slow down the age-related decline of cognition. The aim of this review is to summarise and assess the role of combined physical-and-cognitive-training characteristics in improving cognitive performance and to propose an effective training scheme within the frame of a suitable experimental design.

**Methods:**

A systematic electronic literature search was conducted in selected databases. The following criteria were compulsory for inclusion in the study: 1. A (Randomized) Controlled Trial (RCT or CT) design; 2. Implementation of combined physical and cognitive training, either simultaneously (dual task) or subsequently - at least one hour per weekly over four weeks or more; 3. Cognitive outcomes as a study’s endpoint.

**Results:**

Twenty articles met the inclusion criteria. It appears that either simultaneous or subsequently combined physical and cognitive training is more successful compared to single physical or single cognitive exercise. Training characteristics like length, frequency, duration, intensity and level of task difficulty seem to determine cognitive performance. However, the articles show that cognitive improvement seems to remain somewhat confined to trained cognitive functions rather than generalising to other cognitive or daily-living skills.

**Conclusion:**

Due to methodological heterogeneity among studies, results need to be treated with caution. We critically discuss the role of training characteristics and propose a potentially effective training intervention within an appropriate experimental design.

## Background

Daily activities often require simultaneous performance of two tasks (dual-tasking). Due to their complexity and high demands in motor and cognitive resources, they are difficult to perform, especially by older adults and people with brain pathology [1–3]. Training physical and/or cognitive skills related to daily activities seems to result in ameliorated physical and mental abilities [4]. Physical exercise like cardiovascular or strength training improves balance, endurance, flexibility, and strength, as well as cognition (attention, executive function, etc.) through a series of biological and neural mechanisms, including change of metabolic (oxygen, glucose) and neurochemical (dopamine, neurotrophines) activity in the brain [5–9]. Single cognitive training has also been shown to induce improvements in the targeted cognitive functions, whether visuospatial working and episodic memory, executive function, or speed of information processing [10–20]. Combined physical and cognitive exercise in the form of simultaneous (dual-tasking) or subsequent training seems, however, to render better results in cognitive performance than either type of single training alone [8, 21–23].

To the best of our knowledge, training parameters of combined physical and cognitive exercise (both dual-tasking and subsequent training) that contribute to the improvement of cognitive performance have not been extensively evaluated and summarised in the frame of a comprehensive review paper until now. We undertook the present study in order to clarify the prerequisites of a training that is effective in terms of improving physical and cognitive performance. We investigated the type of physical and cognitive training that brings about the most significant cognitive improvements, as well as what the required length (minutes (min.) per session), frequency (sessions per week), and duration (number of weeks) of this training should be, and we propose an experimental design that integrates these training prerequisites.

## Methods

### Definitions of training

For the purpose of this study we considered physical exercise as a planned, structured, and repetitive activity for a set period of time in order to maintain or improve the physical condition of a person [24, 25]. We considered cognitive training as a process of systematic and planned practice of cognitive functions with the aim of sustaining or enhancing cognitive performance and/or improving everyday-living skills [26]. It makes use of “challenging” cognitive tasks, i.e. tasks demanding enough so that one cannot solve them at once (understimulation), but still appropriate for one’s cognitive level in order to avoid frustration from constant failure. A combined intervention should include a stimulating physical training with a gradually increasing level of difficulty, as previously described [27–29], as well as cognitive training, conducted either simultaneously in the form of dual task interventions (I-DT) or subsequent training interventions (I-S).

### Search strategy

A systematic electronic search of literature was carried out online through Ovid MEDLINE, Ovid EMBASE, and Web of Science databases published between 2002 and 2015. The search strategy was conducted via the various databases by using a keyword search of the following Medical Subject Headings (ME.S.H.) terms: (“dual-task*” OR “dual-task training” OR “dual-task intervention” OR “combine*”) AND (“physical training” OR “exercise”) AND (“cognitive training” OR “cognition” OR “mental”) AND (“random*” OR “controlled trial”). In addition, a list of references including relevant original studies or reviews was also scanned for additional bibliography. Only studies published in English were considered.

### Selection process and data extraction

This paper follows the *PRISMA Statement* guidelines for review articles [30]. All articles retrieved until June 30, 2015 were separately screened by title, abstract, and relevance by two reviewers, namely AL and AI. Articles that were found to be irrelevant were discarded. Full texts were only taken into consideration if the studies seemed to be relevant for inclusion. The following inclusion criteria were implemented: (a) RCT or CT design (b) combined physical and cognitive intervention (performed either simultaneously or subsequently) with a frequency of at least one session per week over four weeks or more, which has been shown to be the minimum frequency necessary for the training to take effect [28], (c) cognitive outcomes as an endpoint. Studies were excluded if they were: (a) review articles or meta-analyses, (b) non-intervention trials, (c) non-English-language papers. There was no restriction with respect to the mean age and health condition of the sample included in the studies. Disagreements on inclusion were resolved by a third party (BT) [31]. Data extraction was performed independently by the same two reviewers (AL, AI) using a standardised form. The following data were extracted from the included articles: (a) study design, total sample size, number of group participants, gender ratio, level of education, health condition and method of recruitment (i.e., e-mail, community, university, retirement home, etc.); (b) characteristics of single and combined physical and cognitive training (duration, length, frequency, and intensity), handling of the control group; (c) both short- and long-term-effects of single and combined training on cognition and daily-life activities.

### Evaluation of methodological quality

A qualitative evaluation of the included studies took place. The *Physiotherapy Evidence Database* (PEDro) scale [32] was used to assess methodological aspects of the studies according to 11 criteria. This rating system enabled the quality of the studies to be assessed free of bias. A study gets one point for every fulfilled criterion and zero points for non-fulfilled criteria. A total score of nine points or more indicates a high level of methodological quality, whereas scores between 6 and 8 show a medium quality. Scores of six points or less represent a low level of methodological quality. Disagreements on rating between the two reviewers (AL, AI) were settled by a third party (BT).

## Results

**Fig. 1:**
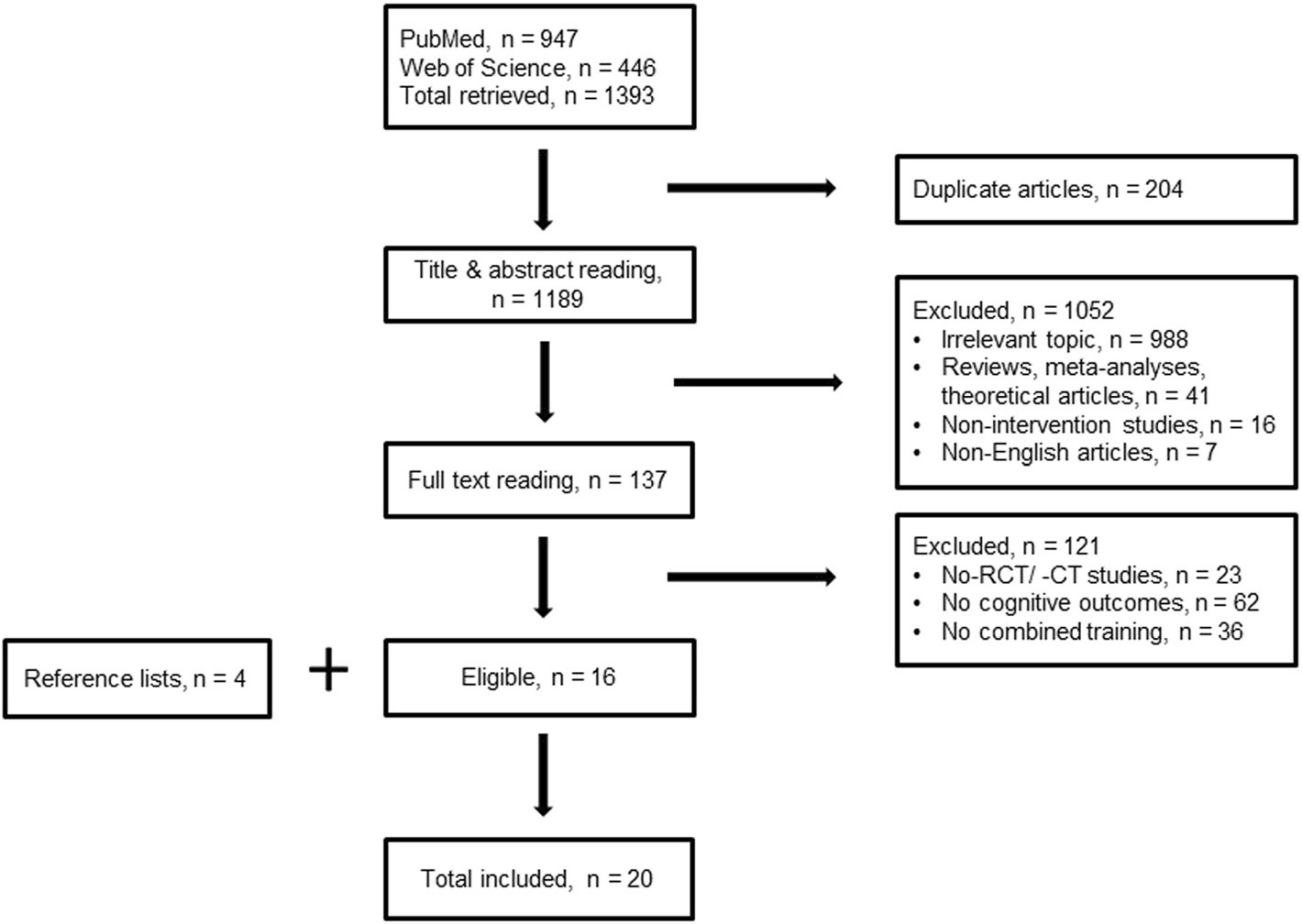
Process of studies’ selection

A flow-chart of the selection process is illustrated in Fig. 1. The database search retrieved a total of 1393 likely relevant articles. Among them 204 were discarded as duplicates. After screening the remaining articles by abstract and title 1052 were excluded due to topic irrelevance, review, meta-analytic or theoretical orientation, implementation of a non-intervention study design or use of a language other than English. A total of 137 articles were considered as full text. Among them 121 were excluded, as they failed to meet the required inclusion criteria. The remaining 16 articles were evaluated as eligible for inclusion. Four additional studies found in the reference list of relevant systematic reviews or meta-analyses were regarded as relevant for inclusion as well, making a total of 20 articles included in the review.

The year of publication of the included articles ranged from 2002 to 2015. However the most frequent publication year was 2013 (*n* = 6 studies). A RCT study design was adopted by 15 studies (see Table 1). The sample size of the studies ranged between *n* = 13 [32] and *n* = 375 [33]. Average age of participants ranged from 44.4 [34] to 82.3 years [35]. Cognitively healthy participants were recruited in 14 studies (see Table 1). Two studies included people with minor cognitive complaints [34, 36], one recruited patients with stroke pathology [37], while three studies examined patients with dementia or Alzheimer’s disease [35, 38, 39]. The total intervention time ranged between 175 min. (7–14 min. per session five times weekly i.e., at least 35 min. per week for five weeks [34]) and 72 h (120 min. three times weekly for 12 weeks [35], see Tables 2 and 3).

**Table 1:** Description of studies’ characteristics

Study	Design	Participants					
		Total sample (Male/Female)	Intervention group *n* (mean age ± SD)	Comparison and control groups *n* (mean age ± SD)	Health condition	Recruitment	Education
Simultaneous Intervention (Dual Task)							
Choi et al., 2015 [37]	RCT	*N* = 21 Analysed *n* = 20 (12/8)	I-DT: *n* = 10 (64.8 ± 10.5 years.)	SPT: *n* = 10 (54.6 ± 11.8 years.)	S	Hospital	N.I.
Coelho et al., 2013 [38]	CT	*N* = 27 (N. I.)	I-DT: *n* = 14 (78.0 ± 7.3 years.)	NA-C: *n* = 13 (77.1 ± 7.4 years.)	AD	Community	I-DT: 5.8 ± 3.8 years. NA-C: 3.7 ± 2.3 years.
De Andrade et al., 2013 [39]	CT	*N* = 30 (6/24)	I-DT: *n* = 14 (78.6 ± 7.1 year.)	NA-C: *n* = 16 (77.0 ± 6.3 years.)	AD	Community	I-DT: 5.1 ± 2.9 years. NA-C: 3.9 ± 2.5 years.
Evans et al., 2009 [34]	RCT	*N* = 19 (17/2)	I-DT: *n* = 10 (44.4 ± 8.5 years.)	NA-C: *n* = 9 (45.1 ± 9.7 years.)	C	N.I.	N.I.
Hars et al., 2013 [42]	RCT	*N* = 134 (5/129)	I-DT: *n* = 66 (75.0 ± 8.0 year.)	NA-C: *n* = 68 (76.0 ± 6.0 year.)	H	Community	Total *N*: Prim. School: 14.9 % Middle school: 67.2 % High school: 17.9 %
Hiyamizu et al., 2012 [43]	RCT	*N* = 43 Analysed *N* = 36 (10/26)	I-DT: *n* = 17 (72.0 ± 5.1 year.)	SPT: *n* = 19 (71.2 ± 4.4 years.)	H	Community	N.I.
Kayama et al., 2014 [44]	CT	*N* = 48 (N.I.) Analysed *n* = 41	I-DT: *n* = 26 (N.I.)	SPT: *n* = 15 (N.I.)	H	Community	N.I.
Marmeleira et al., 2009 [45]	RCT	*N* = 32 (25/7)	I-DT: *n* = 16 (68.2 ± 6.5 years.)	NA-C: *n* = 16 (68.4 ± 6.7 years.)	H	Community	I-DT: 4.8 ± 3.1 year. NA-C: 5.1 ± 2.2 years.
Plummer-D’Amato et al., 2012 [41]	Pilot RCT	*N* = 17 (1/16)	I-DT: *n* = 10 (76.6 ± 5.6 years.)	SPT: *n* = 7 (76.7 ± 6.0 year.)	H	Local senior centre	I-DT: 12.6 ± 2.5 years. STG: 12.9 ± 2.3 years.
Schwenk et al., 2010 [35]	RCT	*N* = 61 (22/39) Analysed *N* = 49	I-DT: *n* = 26 (80.4 ± 7.1 year.)	A-C: *n* = 35 (82.3 ± 7.9 years.)	D	Hospital	I-DT: median 11 year. A-C: median 11 year.
Theill et al., 2013 [46]	CT	*N* = 63 (17/46^a^) Analysed *N* = 51	I-DT: *n* = 18 (72.4 ± 4.2 years.)	SCT: *n* = 12 (73.3 ± 6.1 year.) NA-C: *n* = 21 (70.9 ± 4.8 years.)	H	Community and participant pool of the University of Zurich	I-DT: 13.8 ± 3.0 year. SCT: 14.9 ± 4.9 years. NA-C: 13.2 ± 2.9 years.
Yokoyama et al., 2015 [47]	RCT	*N* = 27 Analysed *N* = 25 (2/23)	I-DT: *n* = 12 (74.2 ± 4.3 years.)	SPT: *n* = 13 (74.2 ± 3.4 years.)	H	Community	I-DT: 11.9 ± 1.7 years. STG: 12.0 ± 1.8 years.
You et al., 2009 [40]	RCT	*N* = 13 (2/11)	I-DT: *n* = 8 (70.5 ± 6.8 years.)	A-C: *n* = 5 (68.0 ± 3.3 years.)	H	Local community centres	N.I.
Subsequent Intervention							
Barnes et al., 2013 [36]	RCT	*N* = 126 (47/79)	I-S: *n* = 32 (74.8 ± 6.1 year.)	SCT: *n* = 31 (73.8 ± 5.7 years.) SPT: *n* = 31 (71.1 ± 5.5 years.) A-C: *n* = 32 (73.9 ± 6.3 years.)	C	Community	I-S: 16.7 ± 2.2 years. SCT: 16.8 ± 2.3 years. SPT: 15.6 ± 2.8 years. A-C: 16.3 ± 2.1 year.
De Bruin et al., 2013 [48]	RCT	*N* = 16 (5/11) Analysed *N* = 13 (5/8)	I-S: *n* = 6 (79.8 ± 6.8 years.)	SPT: *n* = 7 (75.0 ± 8.3 years.)	H	Assisted living facility	N.I.
Fabre et al., 2002 [49]	RCT	*N* = 32 (5/27)	I-S: *n* = 8 (64.9 ± 1.4 years.)	SCT: n = 8 (67.5 ± 1.2 years.) SPT: *n* = 8 (65.4 ± 2.2 years.) A-C: *n* = 8 (65.7 ± 1.5 years.)	H	Clubs	I-S: 12.1 ± 1.2 years. SCT: 12.7 ± 1.2 years. SPT: 11.2 ± 1.3 years. A-C: 12.1 ± 1.4 years.
Legault et al., 2011 [50]	RCT	*N* = 73 (36/37)	I-S: *n* = 19 (76.9 ± 4.0 year.)	SCT: *n* = 18 (76.0 ± 5.2 years.) SPT: *n* = 18 (77.5 ± 4.8 years.) A-C = 18 (75.4 ± 4.8 years.)	H	Community	Higher than High School I-S: 68 % SCT: 78 % SPT: 83 % A-C: 72 %
Oswald et al., 2006 [33]	CT	*N* = 375 (132/243), Age: 75–93 (79.5 ± 3.5) Analysed *N* = 179^a^ at a five-year follow-up	I-S: *n* = 32, *n* = 17^a^	SCT: *n* = 57, *n* = 29^a^ SPT: n = 32, *n* = 15^a^ SPT + PE: *n* = 36, *n* = 18^a^ PE: n = 115, *n* = 47^a^ NA-C: *n* = 103, *n* = 53^a^	H	Community	Total *N*: Prim. School: 41.1 % Sec. school: 39.2 % “Abitur”: 14.4 % University: 5.3 %
Shatil, 2013 [51]	RCT	*N* = 122 (38/84)	I-S: *n* = 29 (79 ± 5.5 years.)	SCT: *n* = 33 (80 ± 5.4 years.) SPT: *n* = 31 (79 ± 5.8 years.) A-C: *n* = 29 (81 ± 5.3 years.)	H	Retirement village	Total *N*: 15.7 ± 2.43 years.
Van het Reve et al., 2014 [52]	RCT	*N* = 182 Analysed *N* = 156 (55/101)	I-S: *n* = 74 (81.1 ± 8.3 years.)	SPT: *n* = 82 (81.9 ± 6.3 years.)	H	Community and Local senior centre	Total *N*: University/College:7.1 % Vocational Education: 59.6 % No education: 26.3 %

*N* total number of study sample, *n* number of group participants, *yrs.* years of age, *SD* standard deviation, *RCT* randomised controlled trial, *CT* controlled trial, *I-DT* simultaneous physical and cognitive training Intervention (Dual Task) group, *I-S* subsequent physical and cognitive training Intervention group, *SCT* single cognitive training, *SPT* single physical training, *PE* psycho-educational training, *A-C* active control, *NA-C* non-active Control, *H* healthy/ cognitively healthy, *C* cognitive complaints/ mild impairment, *S* subacute stroke, *D* dementia; *AD* Alzheimer’s Disease, *N. I.* no Information available

^a^Remaining participants after exclusion of drop-outs, no information about their gender ratio

**Table 2:** Characteristics of simultaneous and subsequent interventions, comparison and control condition

Study	Combined physical and cognitive training intervention		Comparison groups	Control group
	Physical part	Cognitive part		
Simultaneous Intervention (Dual Task)				
Choi et al., 2015 [37]	30 min × 5 times per week for 4 weeks– Total intervention time: 600 min (10 h)		SPT	-
	Physical therapy, balance-training	Memory, learning ability		
Coelho et al., 2013 [38]	60 min × 3 times per week for 16 weeks with an increasing level of difficulty – Total intervention time: 2880 min (48 h)		-	NA-C
	Training of aerobic capacity (65–75 % of max. heart rate for age), resistance- flexibility- and balance- and agility-training Measured: Heart rate, blood pressure	Attention, executive function, psychomotor ability, verbal fluency		
De Andrade et al., 2013 [39]	60 min × 3 times per week for 16 weeks with an increased level of difficulty – Total intervention time: 2880 min (48 h)		-	NA-C
	Training of aerobic capacity (65–75 % of max. heart rate), strength- flexibility- and balance-training Measured: Heart rate	Working memory and verbal fluency		
Evans et al., 2009 [34]	7–14 min × 5 times per week for 5 weeks – Total intervention time: 175–360 min (3–6 h)		-	NA-C
	Walking vividly for 2 min	Divided attention and meta-attention		
Hars et al., 2013 [42]	60 min once a week for 25 weeks - Total intervention time: 1500 min (25 h)		-	NA-C
	Multitask exercises (walking, handling objects, quick reactions) following music	Frontal-lobe cognitive function		
Hiyamizu et al., 2012 [43]	60 min × 2 times per week for 12 weeks – Total intervention time: 1440 min (24 h)		SPT	-
	Walking, strength- and balance-training	Working memory, visual scanning and verbal fluency		
Kayama et al., 2014 [44]	75–80 min once a week for 12 weeks - Total intervention time: 900–960 min (15–16 h)		SPT	-
	Training of aerobic capacity, strength, balance and flexibility	Arithmetical reasoning-ability		
Marmeleira et al., 2009 [45]	60 min × 3 times per week for 12 weeks – Total intervention time: 2160 min (36 h)		-	NA-C
	Training of aerobic capacity, walking	Visual attention, executive function, speed of information processing, psycho-motor performance		
Plummer-D’Amato et al., 2012 [41]	45 min × 1 time per week for 4 weeks – Total intervention time: 180 min (3 h)		SPT	-
	Gait, balance and agility training	Working memory, verbal learning, verbal fluency		
Schwenk et al., 2010 [35]	120 min × 2 times per week for 12 weeks – Total intervention time: 2880 min (48 h)		-	A-C
	Progressive resistance- and functional balance training	Working memory		
Theill et al., 2013 [46]	30 min × 2 times per week for 10 weeks – Total intervention time: 600 min (10 h)		SCT	NA-C
	Walking on treadmill Measured: Heart rate	Working memory		
Yokoyama et al., 2015 [47]	60 min × 3 times per week for 12 weeks – Total intervention time: 2160 min (36 h)		SPT	-
	Training of aerobic capacity, resistance- and flexibility-training	Arithmetic or word tasks, switch walking direction		
You et al., 2009 [40]	30 min × 5 times per week for 6 weeks (total of 18 sessions/participant) – Total intervention time: 540 min (9 h)		-	A-C
	Walking a 30 m walkway	Verbal episodic memory, working memory		
Subsequent Intervention				
Barnes et al., 2013 [36]	60 min physical training + 60 min cognitive training – Total: 120 min × 3 times per week for 12 weeks – Total intervention time: 4320 min (72 h)		SCT SPT	A-C
	Training of aerobic capacity and strength, stretching, relaxation Measured: Heart rate	Divided attention, working memory, visual and auditory perception		
De Bruin et al., 2013 [48]	45 min physical training × 2 times per week for 12 weeks + 10 min cognitive training × 3–5 times per week for 10 weeks – Total intervention time: 1380–1578 min (23–26,3 h)		SPT	-
	Training of aerobic capacity (e.g. stair climbing, etc.), strength and balance	Attention (alertness, selective, divided)		
Fabre et al., 2002 [49]	60 min physical training × 2 times per week for 8 weeks + 90 min cognitive training × 1 time per week for 8 weeks – Total intervention time: 1680 min (28 h)		SCT SPT	A-C
	Training of aerobic capacity (e.g. jogging) Measured: Heart rate	Attention, episodic and working memory, verbal learning, verbal fluency, visual/auditory perception		
Legault et al., 2011 [50]	60 min physical training (at experimental facility) × 2 times per week plus 30 min at home × 1-2 times per week: 150–180 min per week for 16 weeks + 40–48 min cognitive training × 2 times per week for 8 weeks and afterwards 40–48 min once weekly for 8 weeks – Total intervention time: 3360–4032 min (56–67,2 h)		SCT SPT	A-C
	Training of aerobic capacity and flexibility, walking, cycling	Verbal learning, episodic memory		
Oswald et al., 2006 [33]	45 min physical training + 90 min cognitive training once weekly for 30 weeks - Total intervention time: 4050 min (67,5 h)		SCT SPT PE SPT + PE	NA-C
	Flexibility- and balance-training, motor coordination (gymnastic exercises, dancing)	Attention, episodic memory, speed of information processing		
Shatil, 2013 [51]	45 min physical training + 40 min cognitive training × 3 times per week for 16 weeks – Total intervention time: SPT = 2160 min (36 h), SCT = 1920 min (32 h)		SCT SPT	A-C
	Training of aerobic capacity, strength and flexibility Measured: Heart rate	Attention, memory, perception, verbal learning, executive function, speed of information processing and motor coordination		
Van het Reve et al., 2014 [52]	40 min physical training × 2 times per week + 10 min of cognitive training 3 × times per week for 12 weeks – Total interventin time: SPT = 960 min (16 h), SCT = 360 min (6 h)		SPT	-
	Strength- and balance-training	Attention (alertness, selective, divided)		

*A-C* active control group, *NA-C* non-active control group, *SCT* single cognitive training, *SPT* single physical training, *PE* psycho-educational training, *h* hours, *min* minutes, *-* non-existent

**Table 3:** Short and long term effects of simultaneous and subsequent training on cognitive performance and everyday living skills

Study	Treatment condition		Outcome measure of interest	Targeted cognitive function	Everyday living skills	Long term effects on cognition
Simultaneous Intervention (Dual Task)						
Choi et al., 2015 [37]	I-DT	150 min per week for 4 weeks	MMSE, K-MBI, CNT	+	n.e.	n.e.
	SPT	150 min per week for 4 weeks		-		
Coelho et al., 2013 [38]	I-DT	180 min per week for 16 weeks	MMSE, CDT, FAB, PS-WAIS	+	n.e.	n.e.
	NA-C	Daily routine^c^		n.e.		
De Andrade et al., 2013 [39]	I-DT	180 min per week for 16 weeks	MMSE, CDT, FAB, PS-WAIS	+	n.e.	n.e.
	NA-C	Daily routine^c^		n.e.		
Evans et al., 2009 [34]	I-DT	35–70 min per week for 5 weeks	DADT, The Memory Span & Tracking task, Telephone Search with Countning-TEA, DTQ	+	n.e.	n.e.
	NA-C	Daily routine^c^		n.e.		
Hars et al., 2013 [42]	I-DT	60 min per week for 25 weeks	MMSE, CDT, FAB	+	n.e.	n.e.
	NA-C	Daily routine^c^		-		
Hiyamizu et al., 2012 [43]	I-DT	120 min per week for 12 weeks	TMT A & B, Stroop	+	n.e.	n.e.
	SPT	120 min per week for 12 weeks		-		
Kayama et al., 2014 [44]	I-DT	80 min per week for 12 weeks	TMT A & B, a verbal fluency task	+	n.e.	n.e.
	SPT	75 min per week for 12 weeks		-		
Marmeleira et al., 2009 [45]	I-DT	180 min per week for 12 weeks	MMSE, Stroop, TMT B, UFOV, a reaction time test	+	n.e.	n.e.
	NA-C	Daily routine^c^		n.e.		
Plummer-D’Amato et al., 2012 [41]	I-DT	45 min per week for 4 weeks	MoCA, Shipley Vocabulary Test, spontaneous speech, alphabet recitation, a coin transfer task	-	n.e.	n.e.
	SPT	45 min per week for 4 weeks		-		
Schwenk et al., 2010 [35]	I-DT	240 min per week for 12 weeks	MMSE, CERAD battery, TMT A & B, serial S2 forward- and S3 backward-test	+	n.e.	n.e.
	A-C	120 min per week for 12 weeks		n.e.		
Theill et al., 2013 [46]	I-DT	60 min per week for 10 weeks	MMSE, Computer-based tasks, n-back task, counting backwards	+	n.e.	n.e.
	SCT	60 min per week for 10 weeks		+		
	NA-C	Daily routine^c^		n.e.		
Yokoyama et al., 2015 [47]	I-DT	180 min per week for 12 weeks	MMSE^+,^ TMT	+	n.e.	n.e.
	SPT	180 min per week for 12 weeks		-		
You et al., 2009 [40]	I-DT	150 min per week for 6 weeks	MMSE, a word memorizing task, arithmetic calculations	+	n.e.	n.e.
	A-C	150 min per week for 6 weeks		n.e		
Subsequent Intervention						
Barnes et al., 2013 [36]	I-S	360 min per week for 12 weeks	RAVLT, TMT A & B, DSST, EFT, UFOV, a verbal fluency task	+^a^	n.e.	n.e.
	SCT	180 min per week for 12 weeks		+^a^		
	SPT	180 min per week for 12 weeks		+^a^		
	A-C	180 min per week for 12 weeks		n.e.		
De Bruin et al., 2013 [48]	I-S	90 min per week for 12 weeks + 30–50 min/week for 10 weeks	MMSE, Reaction time tasks	+	n.e.	n.e.
	SPT	90 min per week for 12 weeks		+^b^		
Fabre et al., 2002 [49]	I-S	210 min per week for 8 weeks	WMS, BEC 96 Questionnaire	+	n.e.	n.e.
	SCT	90 min per week for 8 weeks		+		
	SPT	120 min per week for 8 weeks		+		
	A-C	120 min per week for 8 weeks		n.e.		
Legault et al., 2011 [50]	I-S	230–276 min per week for 8 weeks + 190–228 min/week for 8 weeks	Hopkins VLT, Logical Memory Task-WMS-III, Self-Ordered Pointing Task, 1-Back and 2-Back tasks, EFT, Task Switching Test, TMT A & B	-	n.e.	n.e.
	SCT	80–96 min per week for 8 weeks + 40–48 min/week for 8 weeks		-		
	SPT	150–180 min per week for 16 weeks		-		
	A-C	1 × per week for 16 weeks		n.e.		
Oswald et al., 2006 [33]	I-S	135 min per week for 30 weeks	WAIS, NAI	+	+	+
	SCT	90 min per week for 30 weeks		+	-	+
	SPT	45 min per week for 30 weeks		-	-	-
	PE	90 min per week for 30 weeks		-	-	-
	SPT + PE	135 min per week for 30 weeks + PE		-	+	-
	NA-C	Non-active (no detailed information)		n.e.	n.e.	n.e.
Van het Reve, et al., 2014 [52]	I-S	70 min per week for 12 weeks	TMT A & B, VTS, a reaction time task	+	n.e	n.e.
	SPT	40 min per week for 12 weeks		-		
Shatil, 2013 [51]	I-S	255 min per week for 16 weeks	MMSE, CogniFit training Programme	+^a^	n.e.	n.e.
	SCT	120 min per week for 16 weeks		+^a^		
	SPT	135 min per week for 16 weeks		-		
	A-C	60 min per week for 16 weeks		n.e.		

*I-DT* simultaneous physical and cognitive training intervention (Dual Task), *I-S* subsequent physical and cognitive training Intervention, *SPT* single physical training, *SCT* single cognitive training, *PE* psycho-educational training, *A-C* active control group, *NA-C* non active control group, *min* minutes; + = significant effect (*p* < 0.05); - = no effect (*p* > 0.05), *n.e.* not estimated

*MMSE* mini-mental state examination (^+^ modified Mini-Mental State Examination-3MS), *K-MBI*, Korean modified barthel index for daily activities, *CNT* computerized neuropsychological test, *FAB* frontal assessment battery, *CDT* Clock Drawing Test, *PS (WAIS)* symbol search subtest (Wechsler Adult Intelligence Scale), *DADT* divided attention and dual-tasking battery, *TEA* test of everyday attention, *DTQ* dual-tasking questionnaire, *TMT A & B* trail making test parts A and B, *UFOV* useful field of view, *MoCA* Montreal cognitive assessment, *CSRT* choice stepping reaction time, *CERAD batttery* consortium-to-establish-a-registry-for-Alzheimer’s-disease battery, *RAVLT* ray auditory verbal learning test, *DSST* digit symbol substitution test, *EFT* Eriksen flanker test, *WMS* Wechsler memory scale, *VLT* verbal learning test, *NAI* neuropsychological aging inventory, *VTS* Vienna test system (computerized cognitive assessment)

^a^Training of a wide range of cognitive functions. Improvement found only in them.^b^Significant positive effect found only for one of the two outcomes, ^c^duration same as in intervention group

### Training characteristics

#### Type of physical and cognitive training

All studies included an intervention group, in which combined physical and cognitive training was implemented. Thirteen of them included a dual-task intervention (I-DT), in which simultaneous physical and cognitive training was applied [34, 35, 37–47]. In the rest of the studies (*n* = 7) a subsequent approach (I-S) was employed, in which physical and cognitive training took place consecutively [33, 36, 48–52]. In addition to the combined physical and cognitive training group, these studies included a single physical and/or a single cognitive training group (referred to as “comparison groups” in Table 1).

Fifteen of the included studies used a combination of different types of physical training, while five only one type (four included walking, one jogging). Irrespective of being combined with other types of physical exercise or not, cardiovascular exercise was involved in 16 studies, strength training in ten, balance tasks in nine, and flexibility in seven. Regarding cognitive training, nine studies trained attention, fifteen executive function/ working memory, and five episodic memory, verbal fluency and verbal learning. Four studies considered perception, while three considered speed of information processing, or motor coordination (see Table 2). Eighteen out of all reviewed studies reported improved cognitive performance in the combined-training-group (see Table 3). Among them 17 included aerobic or strength training (or a combination of them) in the physical-training section of the intervention, as well as attention and/or executive function/ working memory training in the cognitive part (see Table 2).

#### Frequency, length and duration of the combined training

The reviewed studies differed in length, frequency, and duration of training. Thirteen [34, 35, 37–47] implemented a simultaneous intervention, in which the length of the physical and cognitive training ranged between 35 to240 min. per week for a period of 4 to 25 weeks (see Table 2). Seven studies [33, 36, 48–52] included a subsequent approach, in which combined training ranged from 70 to 360 min. weekly over a period of 8 to 30 weeks. Altogether four studies implemented a program in which combined training lasted one hour or less per week [34, 41, 42, 46]. In five of the studies training lasted more than 3 h per week [35, 36, 49–51]. However, most of the studies involved a training program of one to three hours weekly for a period of 4 to 30 weeks [33, 37–40, 43–45, 47, 48, 52].

#### Studies’ endpoints

Eighteen of the reviewed studies considered cognitive outcomes, but no daily-life functional skills. They reported post-intervention improvements only in the trained cognitive functions, but no generalised cognitive benefits. However, two studies took also everyday-living abilities into consideration [33, 37]. Choi et al. [37] reported a within-group improvement in every-day living skills in both groups (I-DT, control) after the intervention, but no between-group differences. Oswald et al. [33] found a significant improvement in the I-S and the SPT-PE group (single physical training and psycho-education) but not in the SPT and SCT (single cognitive training) groups (see Table 3). The same authors reported that in a five-year follow up assessment the I-S group showed significant maintenance of cognitive benefits. None of the remaining 19 studies examined the long-term effects of combined physical and cognitive training.

#### Assessment of methodological quality

The results of this evaluation are presented in Table 4. The reviewed studies scored between 3 and 9 points out of 11 based on a system of one-point-per-criterion match. Seventeen of the studies fulfilled six criteria or more, indicating a level of at least medium quality. Three of them reached a score of nine points, designating high methodological quality. These studies fulfilled most of the criteria with the exception of those concerning blinding of participants and therapist [36, 41], or the intention-to-treat, and the percentage of participants from which measures for at least one outcome were obtained [35]. One study [46] met only three criteria, including baseline characteristics, basic statistical measurements, and between-group comparisons. With the exception of this study, the remaining 19 studies described their inclusion criteria. All studies reported a similarity of baseline characteristics as well as at least one key outcome and its variability [33–52]. Fifteen [34–37, 40–43, 45, 47–52] studies implemented a randomised allocation procedure. In eight studies allocation was concealed [34–36, 41, 43, 47, 48, 52]. Five studies considered participants’ blinding [35, 37, 47, 48, 50], two [35, 43] therapist’s blinding, and five [35, 36, 41–43] assessor’s blinding. Twelve studies analysed outcome measures from more than 85 % of participants initially allocated, and 11 studies adopted an intention-to-treat approach. All studies except for two [48, 49] conducted a series of between-group analyses.

**Table 4:** Evaluation of methodological quality of the reviewed studies according to PEDro-Scale (Maher et al., 2003 [32])

Study	Inclusion/exclusion criteria^a^	Randomisation of groups^b^	Concealment^c^	Similarity of baseline characteristics^d^	Blinded participants^e^	Blinded therapist^f^	Blinded assessor^g^	Key Outcome^h^	Intention to treat^i^	Between groups statistics^j^	Mean/Standard deviation^k^	Final score
Barnes et al., 2013 [36]	yes	yes	yes	yes	no	no	yes	yes	yes	yes	yes	9
Choi et al., 2015 [37]	yes	yes	no	yes	yes	no	no	yes	no	yes	yes	7
Coelho et al., 2013 [38]	yes	no	no	yes	no	no	no	yes	yes	yes	yes	6
De Andrade et al., 2013 [39]	yes	no	no	yes	no	no	no	yes	yes	yes	yes	6
De Bruin et al., 2013 [48]	yes	yes	yes	yes	yes	no	no	no	no	no	yes	6
Evans et al., 2009 [34]	yes	yes	yes	yes	no	no	no	yes	yes	yes	yes	8
Fabre et al., 2002 [49]	yes	yes	no	yes	no	no	no	yes	yes	no	yes	6
Hars et al., 2013 [42]	yes	yes	no	yes	no	no	yes	no	yes	yes	yes	7
Hiyamizu et al., 2012 [43]	yes	yes	yes	yes	no	yes	yes	no	no	yes	yes	8
Kayama et al., 2014 [44]	yes	no	no	yes	no	no	no	yes	no	yes	yes	5
Legault et al., 2011 [50]	yes	yes	no	yes	yes	no	no	yes	yes	yes	yes	8
Marmeleira et al., 2009 [45]	yes	yes	no	yes	no	no	no	yes	yes	yes	yes	7
Oswald et al., 2006 [33]	yes	no	no	yes	no	no	no	no	no	yes	yes	4
Plummer-D’Amato et al., 2012 [41]	yes	yes	yes	yes	no	no	yes	yes	yes	yes	yes	9
Schwenk et al., 2010 [35]	yes	yes	yes	yes	yes	yes	yes	no	no	yes	yes	9
Shatil, 2013 [51]	yes	yes	no	yes	no	no	no	no	yes	yes	yes	6
Theill et al., 2013 [46]	no	no	no	yes	no	no	no	no	no	yes	yes	3
Van het Reve et al., 2014 [52]	yes	yes	yes	yes	no	no	no	no	no	yes	yes	6
Yokoyama et al.,2015 [47]	yes	yes	yes	yes	yes	no	no	yes	no	yes	yes	8
You et al., 2009 [40]	yes	yes	no	yes	no	no	no	yes	yes	yes	yes	7

^a^Eligibility criteria were specified; ^b^Participants were randomly allocated to groups, ^c^Allocation to groups was concealed, ^d^The groups were similar at baseline regarding the most important prognostic indicators, ^e^Participants were not aware of the group, in which they were allocated (blinded), ^f^Staff that administered training was not aware (blind) of the group status (intervention-control), ^g^Assessors measuring at least one key outcome were not aware (blind) of the group status, ^h^Measures of at least one key outcome were obtained from more than 85 % of the subjects initially allocated to groups, ^i^All subjects for whom outcome measures were available received the treatment or control condition as allocated or, where this was not the case, data for at least one key outcome were analysed by “intention to treat”, ^j^The results of between-group statistical comparisons are reported for at least one key outcome, ^k^The study provides both point measures and measures of variability for at least one key outcome

Yes = 1 point, no = 0 points

## Discussion

Investigating the influence of combined physical and cognitive training on cognition is a relatively new and interdisciplinary orientation in this research field. Hence, little evidence is currently available on the role of training characteristics in improving cognitive performance. In the present article we review the findings of 20 studies published between 2002 to 2015 that investigated the influence of combined physical and cognitive training on cognition. Results revealed that (constrained to the trained functions) cognitive improvement after (simultaneously or subsequently) combined physical and cognitive training, provided that it met specific requirements of length, frequency, and duration. We conclude that the three aforementioned training characteristics influence to a great extent the effectiveness of the intervention. We discuss the role of these training parameters and propose a fitting experimental design.

### Training characteristics

#### The role of training type

Our research found that a successful training program includes cardiovascular or strength training sessions combined with attention, or executive function/ working memory practice Concerning the physical part of the combined training it seems that both cardiovascular and strength exercises are needed in order for the training to exert a positive influence on cognitive performance. This finding is in line with previous evidence [6, 53]. An important factor to consider when selecting the type of training is the intensity of the exercise. In order for an intervention program to be effective physical training needs an increasing level of difficulty [27–29]. However, excessive intensity should be avoided for health reasons. Monitoring the participant’s heart rate helps to make sure that the intensity of physical stimuli is sufficiently demanding, but at the same time prevents an undesirable overload [8]. It has been proposed that a steady heart rate of 65–80 % of maximum heart rate during cardiovascular or strength training is enough to activate biological mechanisms that mediate physical alterations in the body [54, 55].

It has not yet been fully understood how the type of physical training influences bodily parameters to improve cognitive performance. However, findings suggest a change in the metabolic activity of the brain. Physical exercise causes an uptake in cerebral blood flow which results in increased oxygen and glycose metabolism [56–59]. Improved cognitive performance has also been related to elevated levels of neuroprotective factors, like neurotrophins and especially Brain-Derived Neurotrophic Factor (BDNF) activation [60–62]. BDNF enhances cerebral plasticity, by promoting neurogenesis, cell proliferation, and synaptogenesis in the hippocampus, as well as angiogenesis in other brain areas [59, 63–66]. Moreover elevated dopaminergic activity in basal ganglia prompted by physical activity, as well as high blood concentration of other biomarkers (norepinephrine, lactate, etc.) contribute to improving memory [67, 68]. These neurobiochemical and physiological effects translate into better cognitive performance only under mentally challenging circumstances [53, 69]. That means that the positive influence of physically challenging exercise appears under cognitively demanding conditions, like those triggered in a combined physical and cognitive training.

It is therefore important that cognitive training in a combined intervention be sufficiently demanding in order to improve cognitive performance. All studies we review in this paper included at least one challenging cognitive task, such as training of attention, executive function or working memory. The fact that two of them [41, 50] found no significant improvement in the cognitive performance of the combined-training group demonstrates the complexity of the interaction between physical and cognitive training. In the case of Plummer-D’Amato et al. [41] we would attribute this finding to the low intensity of the training program, meaning that the training may not have been challenging enough to bring about significant improvement, as it included low intensity exercises such as walking, balance and agility training. In the study of Legault et al. [50] the duration of the training program, a parameter that we discuss in the following unit, may have been insufficient to render a significant effect.

#### Frequency, length and duration of an effective combined training

Our findings suggest that a training scheme of 1 to 3 hours weekly for 12 to 16 weeks (or more) is more likely to lead to detectable improvements in cognitive performance than other training schemes. Our results seem to be in accordance with previous findings of Colcombe & Kramer [6], who proposed that three or more weekly sessions of 30 to 45 min. each (that is at least 90 min. per week) over a period of 6 months or more (at least 2160 min. i.e., 36 h of physical training in total) suffice to improve cognition. Those reviewed studies which met the required criteria of duration, frequency and length of training reported a significant improvement in cognitive performance. One study [50] despite fulfilling the recommended length and frequency reported no significant improvement. We attribute this result to the short duration of the training in this study (see Table 3).

Regarding the length of the cognitive part of the combined intervention, we found that even ten hours of cognitive training suffice to induce an improvement in cognitive performance [10, 20, 70, 71]. The effect of the training, as suggested by our results, remains rather constrained to the targeted cognitive functions. In accordance with this, previous literature supports that cognitive training has a positive effect solely on the targeted cognitive function [10, 20, 70, 71].

In relation to the issue of long-lasting effects, previous longitudinal studies failed to detect any maintenance effects [72, 73]. In this paper we review, however, one study [33] which included a follow-up examination and reported a distinguishable cognitive profit 5 years from training. Given that none of the rest of the studies we reviewed included a follow-up examination, we cannot draw any certain conclusions on what the long-lasting effects of combined physical and cognitive training on cognition are and propose that more research on this field be conducted.

### Methodological considerations

The studies reviewed in this paper differ methodologically in many parameters including experimental design, sample size, duration, length, frequency, and intensity of the intervention program, as well as participants’ characteristics, such as age, health condition, psychological and social parameters. To begin with, group size in 13 of the studies was smaller than 30 potentially suggesting questionable statistical power. Only half of them gave detailed information on recruitment, adherence, and compliance rates (drop-out rate, lost at follow-up, etc.). In addition, the reviewed studies implemented either a between-group or a repeated measures design, but only one study [33] considered including a follow-up assessment. Furthermore there is a great variation of the conducted exercise programs among the reviewed studies. This is because each study defines in a slightly different way physical training and cognitive training with regard to training characteristics (duration, length, frequency, and intensity) and type of training (cardiovascular, strength, balance, etc.). Moreover, cognitive assessment procedures and tools differ from study to study meaning that a wide variety of cognitive tasks has been implemented to train and test cognition. Consequently, the results from various tests are not always directly comparable to each other, even if tests are designed to measure the same cognitive function. Another point to consider is the studies’ approach to combined physical and cognitive training. Thirteen studies used dual tasking as a training approach, whereas seven studies implemented a subsequent approach. It has been proposed that dual tasking provides an advantage against subsequent training due to the activation of the cerebellum and the surrounding brain area that facilitate learning [8]. In our review 12 out of 13 studies that implemented dual tasking and 5 out of 6 that followed a subsequent approach reported a significant improvement in cognition. Thus we cannot firmly confirm or reject literature findings. In the study of Legault et al. [50] reviewed in our paper SPT followed SCT, while in that of Oswald et al. [33] participants of the combined group were first trained in SCT and then in SPT for the half of the intervention time, whereas vice versa during the second half. Although Legault et al. [50] reported no positive effects, Oswald et al. found significant improvement in cognition. This may imply that in case combined training is not simultaneous, SPT could be more beneficial if it precedes SCT. This hypothesis could be supported by basic research findings on the physiological changes caused in the brain after exercise (neurogenesis, etc.) that show cognitive improvement [64, 66]. All in all the great variety of the above described methodological parameters that influence studies’ results account for the controversy among findings.

For the literature to be more conclusive, and studies more comparable to each other, research trials should ideally implement a standardised experimental protocol. We propose an experimental design that includes a combined physical and cognitive training group (dual-task or subsequent training), a single physical-training group, a single cognitive-training group, an active control group preoccupied with physical and cognitive tasks of no training value (e.g., stretching, reading, etc.), and a non-active control group continuing their daily routine (no experimental handling). This design facilitates the isolation of the effects of the single training factors on cognition and the evaluation of the dual-task costs. Furthermore, the length of the training should range between 60 and 180 min. per session at a frequency of three times per week over a period of 3 to 4 months or more. The part of the physical training of the program in order to be stimulating enough should include both a cardiovascular and a strength training section and be conducted under constant monitoring of the heart rate. The cognitive part of the intervention should involve the use of standardised tools (cognitive tests) or attention and/or executive function/ working memory tasks, which are adequately challenging to provoke effortful thinking. The level of task difficulty should be tailored to participants’ performance and be gradually advancing, while feedback information should be given, as it has been shown that training accustomed to performance and feedback benefit cognitive performance [23, 74]. In the face of insufficient evidence to indisputably support the effectiveness of physical exercise on cognitive performance in humans [75], the need for a common line in research protocols is imperative, as it would exclude interfering factors of individual studies that influence their results. Using common research protocols leads, therefore, to having comparable results and safer conclusions, as well as facilitates setting and investigating further questions such as what the exact biological substrates are that mediate the cognitive improvement after combined physical and cognitive training.

### Limitations

A first limitation of this review is that we kept the search strategy rather broad using general terms in order to avoid overlooking possibly relevant articles. This led to the retrieval of a great number of studies that had to be sorted through by two reviewers independently via a manual filtering process by reading titles and abstracts. This filtering process is susceptible to bias owing to the human factor. Moreover, there is heterogeneity among the included studies with respect to the experimental design, sample characteristics, training conditions, cognitive tests used, and outcomes. Due to this fact, comparability of studies is limited to some extent, and conclusions need to be treated with caution. Lastly, we concentrated our literature research only on studies published in English. Therefore, it might be possible that certain relevant articles published in other languages have been omitted.

## Conclusions

To conclude, it seems that combined physical and cognitive training has a positive influence on cognition when it meets specific criteria. Cardiovascular and strength training combined with cognitive training of attention and/or executive function/working memory seem to be an integral part of an effective training program. Because of the heterogeneity of studies with respect to a number of vital methodological parameters, our results are to be interpreted with caution. Future research should, therefore, focus on further investigating the role of training characteristics, considering follow-up assessments and conducting larger–scale clinical trials. It is also crucial that clinical issues be taken into account, such as the usefulness of habitual physical exercise, the importance of preventive and rehabilitative training against cognitive decline, or strategies to positively influence the course of a disease.

## Abbreviations

BDNF, brain-derived neurotrophic factor; CT, controlled trial; I-DT, dual task intervention; I-S, subsequent training intervention; ME.S.H., medical subject headings; min., minutes; PE, psycho-educational training; PEDro, physiotherapy evidence database; RCT, randomized controlled trial; SCT, single cognitive training; SPT, single physical training
